# Study on Microdroplets Generation and Detection Method in Four-Way Microfluid Structure (FWMS) by Double Photoresist Method Pulses

**DOI:** 10.3390/mi16111205

**Published:** 2025-10-23

**Authors:** Lele Luo, Lu Zhang

**Affiliations:** 1 Modern Engineering Training Center, Chang’an University, Xi’an 710018, China; lele1103@chd.edu.cn; 2 State Key Laboratory for Manufacturing Systems Engineering, Xi’an Jiaotong University, Xi’an 710049, China; 3 School of Instrumentation Science and Technology, Xi’an Jiaotong University, Xi’an 710049, China

**Keywords:** microdroplets, double photoresist method pulses, time-sequence tracking, four-way microfluid structure (FWMS)

## Abstract

Hundred-micron-sized microdroplets are widely used in microbial culture, chemical investigations and industrial processes. The size, velocity and frequency of microdroplets significantly affect the cultivation and processing effects. The detections of droplets mainly rely on capacitance detection or imaging, but it requires expensive and complex systems for capacitance detection, and high-throughput imaging detections are challenging. In this study, four-way microfluid structure (FWMS) is proposed for microdroplets generation and detection. FWMS, fixed on a 3D-printed holder, is designed to generate microdroplets (100–500 µm), with optical fibers embedded to collect double photoresist method pulses of scattering light by fast-moving microdroplets. The size and volume of the microdroplets are retrieved by tracking the double pulse signal in the time sequence. In the experiments, 50 groups of microdroplets (a total of 10^5^ microdroplets) with size ranging from 100 to 450 µm were generated and detected. Compared with traditional imaging detection, this method has a better sampling rate and detection error of less than 1.42%, which can provide a simple and accurate integrated microfluid system for microdroplet generation and synchronous detection.

## 1. Introduction

The microdroplets with size about several hundred micrometers are widely applied in many research fields [1,2] such as microbial cultivation [3,4,5,6], chemical reactions [7,8,9,10], food processing [11,12], biomedicine, high-throughput assays, reproductive technologies and tissue engineering [13,14,15,16]. Since the microdroplets are in a multiphase flow system, they form a closed system, which enables a clean environment to be maintained inside the microdroplets. Furthermore, they have the advantages of high monodispersity, controllable size and regular shapes, which can provide good living conditions for microorganism’s growth in a closed system without cross-contamination. Microdroplets also have strong mass transfer ability as closed microreactors for chemical reactions to significantly accelerate their reaction rates [17]. On the other hand, for special food structure design and manufacture, a microdroplet’s high monodispersity makes it effective for the encapsulation and controlled release of food-active and functional compounds. The size, velocity and generation frequency of microdroplets are important. The size can firstly determine the reagent amount in chemical reactions [4]. The effect of chemical reactions inside a microdroplet is secondly related to its flowing velocity. In addition, generation frequency controls the throughput of chemical reactions [5,6,7]. Therefore, it is necessary to track the microdroplet’s dynamic states in real time for ensuring chemical reaction quality in microfluid. In future research, advanced and precise biochemical experiments will be carried out by generating droplets quantitatively on demand, such as molecular detection the and synthesis of new materials [18,19,20,21].

In recent years, some dynamic detection methods for microdroplets in microfluid have been studied [22,23,24,25,26,27,28,29,30,31,32,33,34,35,36]. Yang et al. proposed an electrical impedance-based detection method [22] to obtain the size and velocity of microdroplets. This detection method requires the high accuracy of circuit design, and for long-term detection, the measurement consistency is very susceptible to environmental factors [23,24,25,26,27,28,29,30]. In order to improve accuracy, laser-induced fluorescence (LIF) technology was put forward by Ocvirk et al. [31]. Microdroplets were first labeled and then excited by an Argon-ion laser to release fluorescence signals. Due to the labeling technology, the sensitivity and accuracy are well improved. Unfortunately, fluorescence photobleaching, phototoxicity, spectral matching problems and so on limit the applications of LIF. Microdroplet imaging methods are then considered [32,33,34,35]. The video processing-based algorithm for microdroplet imaging was proposed by Basu et al. [36] to obtain their size, velocity and morphology in each video frame. In order to ensure accuracy, the sampling frequency of the imaging detector is important. However, imaging detectors with good performances are too expensive to be applied widely.

In this study, microdroplet generation and real-time detection method in four-way microfluid structure (FWMS) by double photoresist method pulses is proposed. A simple four-way microfluid structure is designed to generate microdroplets with sizes 100–500 µm precisely. Photoresist method pulses of label free microdroplets in microfluid are utilized to obtain their size, velocity, generation frequency and other parameters. In order to track microdroplets with high throughput accurately, double photoresist method pulse time-sequence detection method is designed to collect one dimensional signal with high sampling frequency. Our generation and detection system for microdroplets are integrated in a small and simple device with size 100 mm × 30 mm × 10 mm (length × width × heigh). Furthermore, by analyzing the signals, when Qc = 5.0 mL/h and Qd = 3.0 mL/h, this microdroplet detection system can detect at least 62 samples per second.

## 2. Materials and Methods

### 2.1. System Design

There are four sections in the system of microdroplet generation and detection method in four-way microfluid structure (FWMS) by double photoresist method pulses, which include the inlet, FWMS, microdroplet detection and collection module.

**Figure 1:**
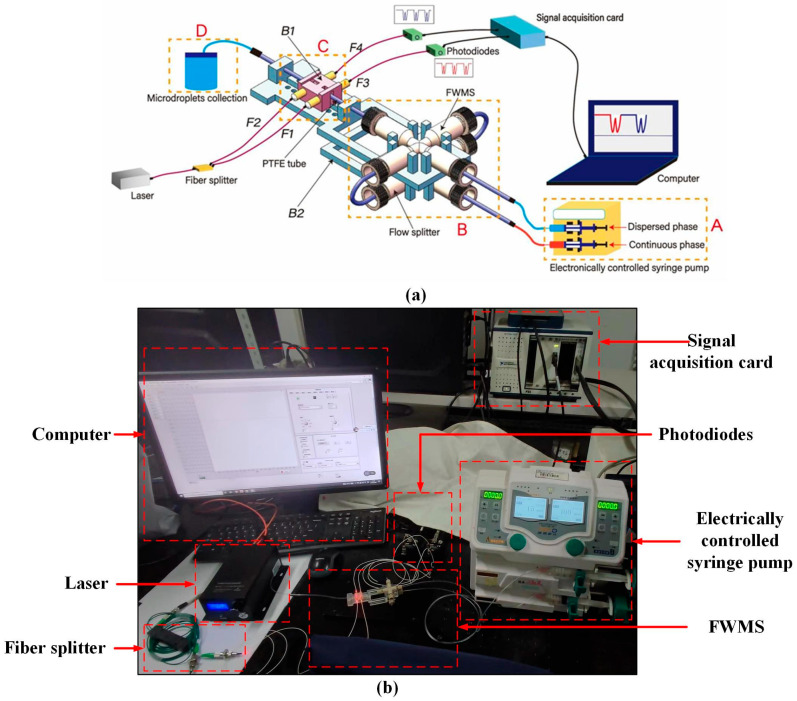
Microdroplet generation and detection system. (**a**) Microdroplet generation and detection system model. (**b**) Photo of the double photoresist method pulse time-sequence detection system.

**Figure 2:**
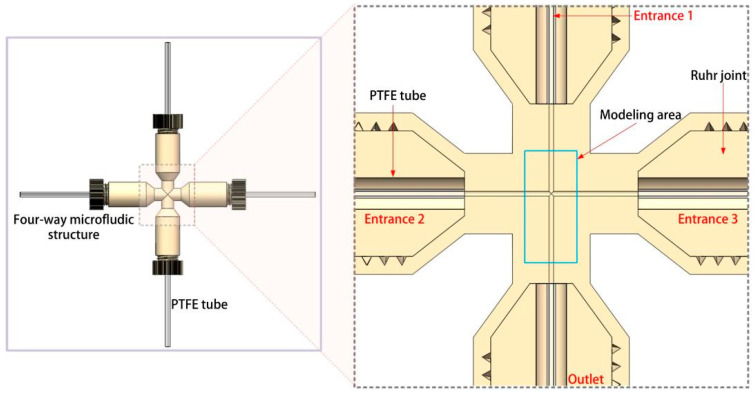
Four-way microfluid structure (FWMS).

In the inlet module (yellow dashed box A in Figure 1), two syringe pumps (Tongsheng Yida Co., Ltd., Xinxiang, China) were used to drive continuous phase and dispersed phase for microdroplet generation. FWMS module (yellow dashed box B in Figure 1) is composed of FWMS and a flow splitter. The detailed structure of FWMS can be found in Figure 2, which shows a cross-shaped microfluid channel with internal diameter of 500 μm. The flow splitter was put below FWMS. It was used to separate the continuous phase from the inlet module into two flows and then injected into FWMS’s Entrance 2 and Entrance 3 in Figure 2. In our experiments, the dimethyl silicone oil as the continuous phase fluid and the water as the dispersed phase were selected. Furthermore, due to the high stability of the fluid in the laminar state, the Reynolds number was kept at a relatively low value. The fluid velocity was approximately between 8.3 mm/s and 26.6 mm/s. To keep the fluid in a laminar state, the channel size was selected as a smaller value. In this study, a PTFE pipe with a diameter of 500 was selected as the microfluidic channel for microdroplet generation and transportation. After calculation, the maximum Reynolds number Re in this study did not exceed 0.014. Therefore, the fluid motion in the experimental conditions of this study satisfied the laminar flow condition. When the appropriate flow rate was set, the microdroplets required for the experiment could be generated.

Four optical fibers (NA = 0.13) with self-focusing lenses (F1 to F4 in Figure 1a, Zhonghui Optoelectronics Technology Co., Ltd., Shenzhen, China) were arranged on both sides of the transparent microchannel. F1 and F2 in the yellow dashed box C of Figure 1a were linked with a semiconductor fiber laser (Beijing Xinglin Ruiguang Technology Co., Ltd. GD3-CC311, Beijing, China) with a wavelength of 650 nm and offering a maximum output power of 2 mW. The incident laser from F1 and F2 was adjusted carefully to focus on the center of the microchannel. A clear resin bracket (B1 in the yellow dashed box C of Figure 1) was prepared by 3D printing technology as a holder for fibers and the microchannel. When a generated microdroplet passed through two focused incident beams, the light scattering phenomenon occurred. The scattering light was collected by photodiodes (Thorlabs Co., Ltd. PDA36A, Newton, NJ, USA) through F3 and F4, respectively, at different times, which provided high-sensitivity light detection within a wavelength range of 350 nm to 1100 nm. The photodiodes were equipped with multi-stage gain adjustment functionality, enabling signal amplification at various levels across 8 discrete steps, each offering a 10 dB increase. With a maximum sampling rate of 10 MHz, they were capable of accurately detecting optical signals as weak as 5 × 10^−12^ W. Double photoresist method pulses of scattering light could be obtained as a time-sequence to record microdroplet passing time through two focused incident beams. The laser, optical fibers with self-focusing lenses (F1 to F4), photodiodes and signal acquisition card (National Instrument Co., Ltd. Pxle-1071, Austin, TX, USA) finally made up the detection module in Figure 1. Visual software was developed by Labview (National Instrument, Inc., Austin, TX, USA) to record time-sequence photoresist method pulses of microdroplets in real time. Another clear resin bracket with size 100 mm × 30 mm × 10 mm (length × width × heigh, B2 in Figure 1) was designed to fix the FWMS and detection module. Finally, in the collection module (the yellow dashed box D in Figure 1), the generated microdroplets were collected by a sealed and sterile reagent dish. Figure 1b shows a photo of the experimental system that has been set up according to the method mentioned.

In Figure 2, the dispersed phase (water) came into FWMS from Entrance 1, and under the shear force of two continuous-phase flow (dimethyl silicone oil; parameters are shown in Table 1) out of Entrance 2 and Entrance 3, the microdroplets in the following polytetrafluoroethylene tube (PTFE tube) of Figure 1a were generated, and their sizes depended on the flow rate of dispersed and continuous phase. A PTFE tube with inner diameter of 500 μm was connected with FWMS as the microchannel.

Throughout microdroplet generation, the fluid in the channel was required to maintain a stable laminar flow regime. In this experiment, the equivalent diameter of the channel was d = 500 μm. At room temperature and atmospheric pressure, the viscosity of water was μ = 0.893 Pa·s. To ensure the fluid remained in a laminar state with a Reynolds number Re < 2100, the maximum fluid injection rate Q should not exceed 642.8 mL/s. The maximum injection speed of the electronically controlled syringe pump used in this study was 100 mL/h. Therefore, the fluid in the microchannel could be maintained in a laminar flow regime during the experiments.

The microdroplet detection module required calibration before operation. At the beginning of the experiment, two acquisition channels corresponding to the photodetector and signal acquisition card were selected in the visualization software. Sampling parameters and ranges were configured accordingly. Once the operation began, signals were displayed in real-time on the software interface as pulse waveforms. Experimental parameters could be adjusted based on the waveforms observed in the visualization software until clear pulse signals were achieved.

### 2.2. Principle

**Figure 3:**
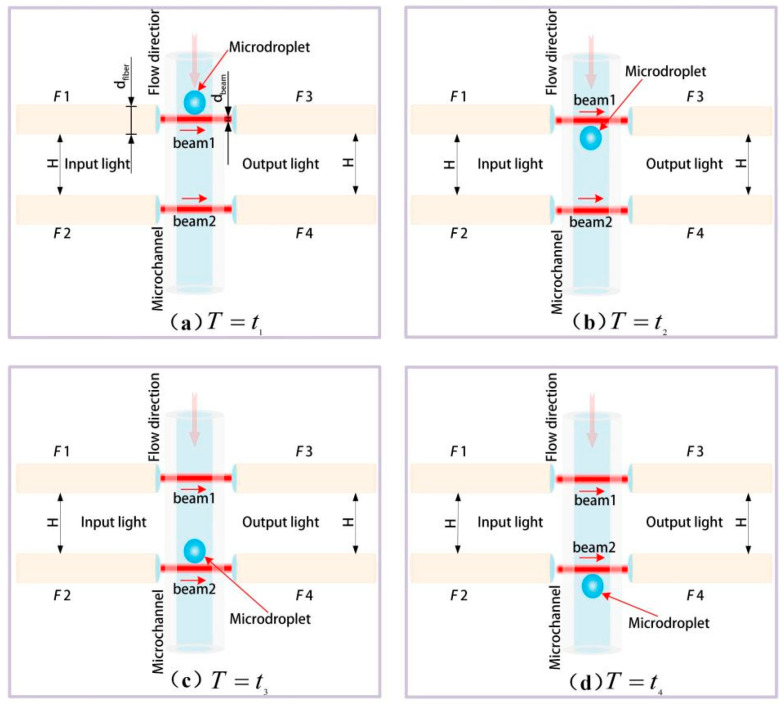
Principle of double photoresist pulses measurement method. (**a**) At *t*_1_, the leading edge of the microdroplet contacts the beam from the first optical fiber. (**b**) At *t*_2_, the trailing edge contacts the same beam. (**c**) At *t*_3_, the leading edge of the microdroplet contacts the beam from the second optical fiber. (**d**) At *t*_4_, the trailing edge contacts the same beam from the second optical fiber.

Double photoresist method pulses are designed to track the microdroplets’ parameters. During time-sequence tracking, there is a correlation between microdroplet’s velocity and size. As shown in Figure 3a,c, let the time be ${T=t}_{1}$ and $T=t_{3}$ when a microdroplet enters the first (beam 1 out of F1 in Figure 3) and the second (beam 2 out of F2 in Figure 3) incident laser beams; when it leaves the first and second beams in Figure 3b,d, the times are $T=t_{2}$ and $T=t_{4}$. The exit pupil diameters of the fibers’ (F1 to F4) self-focusing lenses are $d_{fiber}$ = 3100 μm. Using the self-focusing lens, the incident light from each fiber is focused to a collimated laser beam with a diameter $d_{beam}$ = 100 μm. The distances between the two incident fibers (F1 and F2) and the two collection fibers (F3 and F4) are all set as H = 5000 μm. The flow rates of the dispersed phase (water) and the continuous phase (dimethyl silicone oil) are $v_{d}$ and $v_{c},$ receptively. According to Poisuille’s law, the velocity of fluid along its flowing direction is constant under the laminar flow state. Therefore, in this case, the microdroplet velocity v could be thought equal to that of the continuous phase $v_{c}$, especially in a short measure time; that is, $v=v_{c}$. The velocity v of a microdroplet moving inside a microchannel can be calculated as Equation (1):

(1) $$v=\frac{H+d_{fiber}}{t_{3}-t_{1}}$$

According to $t_{1}$, $t_{2}$, and $t_{3}$, the size of the microdroplet labeled $D^{\prime }$ could be calculated by:

(2) $$D^{\prime }+d_{beam}=v\left(t_{2}-t_{1}\right)=\frac{H+d_{fiber}}{t_{3}-t_{1}}\left(t_{2}-t_{1}\right)$$

Therefore, $D^{\prime }$ can be obtained by Equation (3):

(3) $$D^{\prime }=\frac{H+d_{fiber}}{t_{3}-t_{1}}\left(t_{2}-t_{1}\right)-d_{beam}$$

In addition, according to $t_{1}$, $t_{3}$, and $t_{4}$, the size of the microdroplet labeled as $D^{\prime \prime }$ can also be calculated by:

(4) $$D^{\prime \prime }=\frac{H+d_{fiber}}{t_{3}-t_{1}}\left(t_{4}-t_{3}\right)-d_{beam}$$

The average value D of the microdroplet is calculated by $D=\frac{D^{\prime }+D^{\prime \prime }}{2}$ as the final detection result.

### 2.3. Simulation

FWMS and detection modules are the key sections in the whole system. Therefore, a microdroplet generation simulation for FWMS module and optical simulation for detection module were conducted.

#### 2.3.1. Microdroplet Generation Simulation

**Figure 4:**
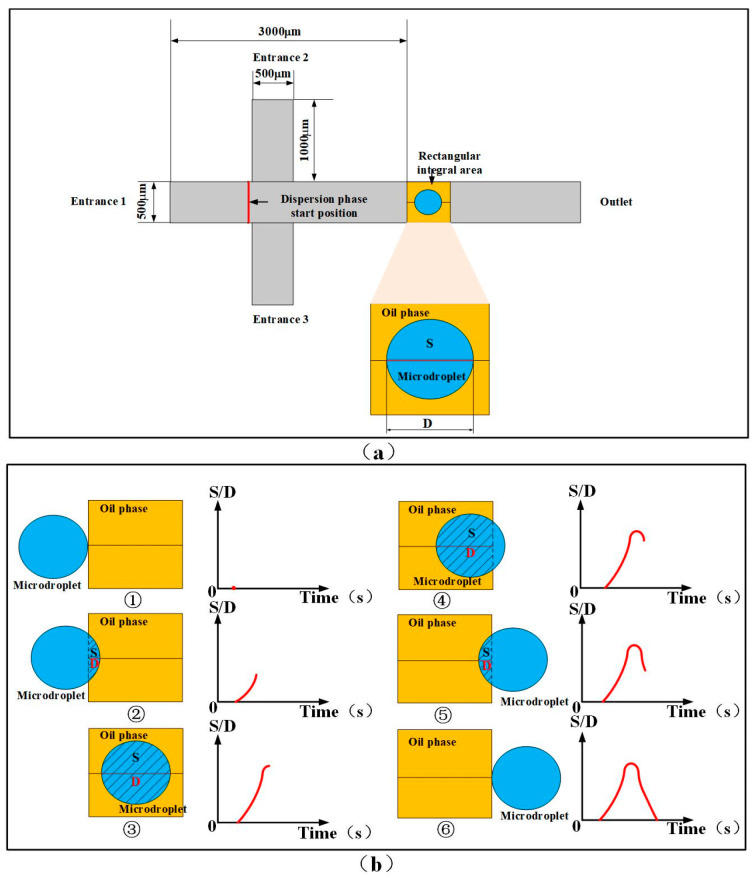
Finite element simulation modeling and integration region. (**a**) Two-dimensional model of FWMS microdroplet generation process, including modeling area and rectangular integral area, and flow rate setting of each phase. (**b**) The droplet generates an analog pulse through a rectangular integral region.

**Figure 5:**
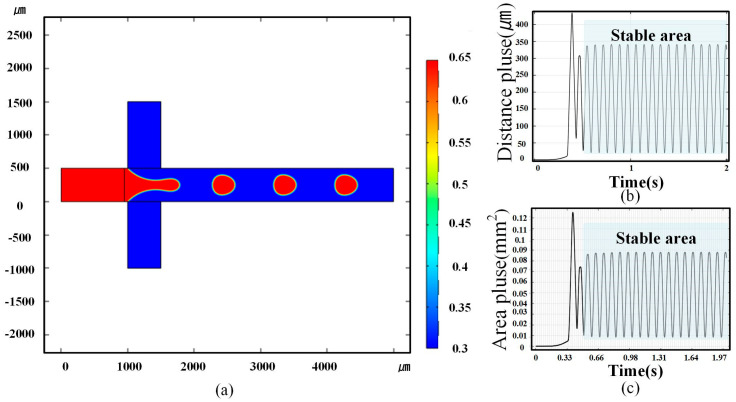
Simulation results of microdroplet generation and integral calculation of microdroplet size and area. (**a**) The simulation results of microdroplet generation. (**b**) The results of microdroplet size integral calculation. (**c**) Integration results of the microdroplet area, and the peak value of the pulse is the area size.

The software of COMSOL Multiphysics 6.0 (COMSOL, Inc., Stockholm, Sverige) was used to simulate the microdroplet generation process, which occurred in the modeling area of the FWMS shown in Figure 4. Before that, parameters were established. Two liquids, water and dimethyl silicone oil, were chosen for preparing microdroplets. The densities were set to 1000 kg/m^3^ for water and 960 kg/m^3^ for the oil (50 CS). The dynamic viscosities were 0.9 × 10^−3^ Pa·s for water and 1 Pa·s for the oil. Other parameters, such as interfacial tension, contact angle and capillary number, were defined according to the material values provided by the software. In the spf interface (laminar flow), the inlet location, the fluid flow velocity, the outlet location and the initial fluid values were set. In the pf interface (phase field), the initial phase values were set. In the multiphysics coupling, the spf and pf modules were primarily coupled so that the phase field evolves within the laminar flow; the velocity field from the spf calculation was used, and the two-phase interface evolution was captured in the pf calculation. Then, the two-dimensional model was meshed to obtain relatively accurate results, using a physics-controlled mesh, with the mesh size set to Ultra-Fine. Components 1 and 2 were meshed separately. The color bar of Figure 5a is the normalized liquid phase coefficient n. The blue color (n = 0) and the red color (n = 1) represent the pure continuous and dispersed phase without dispersion. When n is between 0 and 1, it means the dispersion happens. In total simulation time T=2 s, a series of generated microdroplets’ distance and area pulse results are shown in Figure 5b,c. The amount of generated microdroplets can be counted by pulse number, and the peak value of each pulse represents the size of every generated microdroplet.

**Figure 6:**
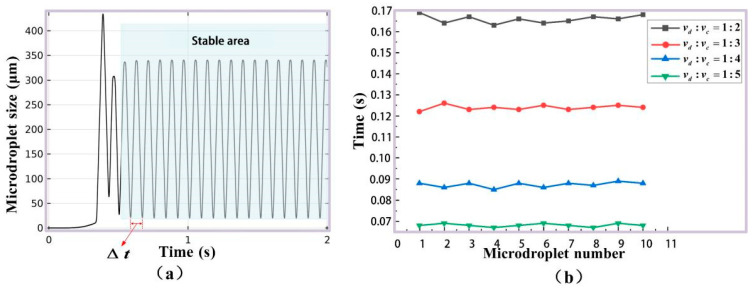
Microdroplet velocity. (**a**) The time ∆*t* for a microdroplet passing through the integration region. (**b**) ∆*t* with different ratios.

From Figure 5b,c, it can be seen that the distance pulse and the area pulse are very uniform, with standard deviations less than 0.5%. The good size consistency of generated microdroplets in simulation can be therefore proven. In order to investigate the velocity and frequency, the time for a microdroplet passing through rectangular integral area was labeled as ∆*t* (Figure 6a), and four groups of simulation experiments were performed with different ratio of $v_{d}$ (the rate of dispersed phase) to $v_{c}$ (the rate of continuous phase), which are $v_{d}:v_{c}$ = 1:2, 1:3, 1:4:1:5. For every simulation, ∆*t* for 10 continuously generated microdroplets was recorded, as shown in Figure 6b. The time error was defined as the maximum ∆*t*, subtracting the minimum one for each simulation. In the four simulations with different ratios of $v_{d}$ to $v_{c}$, the maximum time error was not more than 0.01 s. It is proved that our designed FWMS can generate microdroplets with stable frequency and flow velocity.

The 2D models in Figure 4a were built using COMSOL. With the help of COMSOL component function, two components in Figure 4a were simulated. The first component is the whole modeling area, shown as the gray cross area in Figure 4a. The second component is the rectangular integral area in yellow color. The inside blue circle represents a generated microdroplet. The flow rate of the dispersed phase (water) from Entrance 1 was set as $Q_{d}$, and that of the continuous phase (dimethyl silicone oil) from Entrance 2 and Entrance 3 was $Q_{c}$. When the continuous and dispersed phase were injected into the FWMS with specific flow rates, microdroplets were generated. As they passed through the rectangular integral area, a simulated pulse was generated, as shown in Figure 4b, which could be applied to investigate the microdroplet characteristics. Its abscissa for simulated pulse is the time, and the ordinate is the distance along the diameter direction (named as distance pulse) and the shadow section area (named as area pulse) in Figure 4b, where a microdroplet overlaps with the rectangular integral area. The subfigures ①–⑥ of Figure 4b illustrate a pulse generating process. The microdroplet entering the rectangular integral area starts at the moment of subfigure ① and ends at that of subfigure ⑥. At the moment shown in subfigure ①, the microdroplet is about to come into contact with the rectangular integral area; at the moment shown in subfigure ②, the microdroplet has already made contact with the rectangular integral area; at the moment shown in subfigure ③, the microdroplet has completely entered the rectangular integral area; at the moments shown in subfigures ④ and ⑤, the microdroplet is gradually leaving the rectangular integral area; at the moment shown in subfigure ⑥, the microdroplet has completely left the rectangular integral area. The simulation result with $Q_{d}$ = 1.5 mL/h and $Q_{c}$ = 2 mL/h is given out in Figure 5a as an example. The other parameters are shown in Figure 4a.

As is shown in Figure 5, when a microdroplet passes through the rectangular integral area, a pulse can be generated. The peak value of the pulse depends on the microdroplet’s size, and the number of pulses is the amount of microdroplets. In Figure 5b,c the green area indicates that stably generating region.

#### 2.3.2. Microdroplet Detection Simulation

Double photoresist method pulses time-sequence tracking for the detection module in Figure 1 is simulated by TracePro 2020 software (Lambda Research Corporation, Boston, MA, USA).

**Figure 7:**
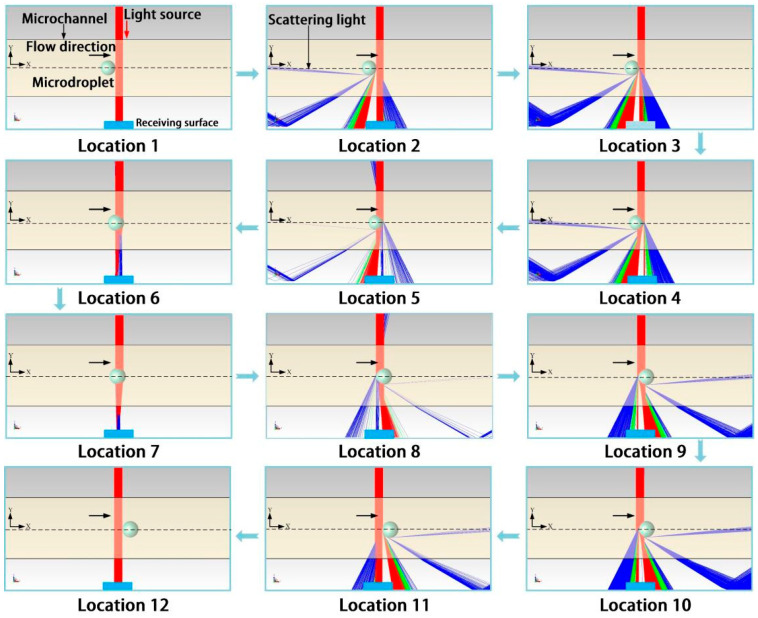
Scatter light of the microdroplets passing through the laser beam.

A Gaussian-distributed lattice light source incident along the Y axis with wavelength 650 nm was selected for the simulation, as shown in Figure 7-Location 1. The centerline of the microchannel, with an inner diameter of 500 μm, was set as the X axis, along which generated microdroplets, with refractive index of $n_{water}$ = 1.33, flowed. The light source and receiving surface were set on both sides of the microchannel’s radial direction, and the area of the receiving surface is set equal to the cross-section area of the optical fiber (F3 and F4 in Figure 1). The refractive index of the continuous phase was $n_{oil}$ = 1.40, and that of the microchannel was $n_{PTFE}$ = 1.35. Location 1 to Location 12 in Figure 7 represent the process of a microdroplet passing through the laser beam. The collected scattering light intensity on the receiving surface during this process was simulated and is shown in Figure 7.

In Figure 7, the different colored lights represent the attenuation of light energy. Red indicates that the light energy has decreased to 100–66% of its original level, green light indicates a decrease to 66–33% of the original level, and blue light indicates a decrease to 33–0% of the original level. The arrow indicates the direction of the flow of the micro droplets. Location 1 and Location 12 of Figure 7 are the situations where the microdroplet does not enter and just leaves the light beam. In this case, the number of light beams collected by the receiving surface is equal and labeled as $m_{1}$. When the microdroplet flowed through the light beam, as shown in Location 2 to Location 11, the scattering phenomenon occurred. Their light beam number collected by the receiving surface is $m_{2}$. The relative intensity I is defined as $I=\frac{m_{2}}{m_{1}}$.

**Figure 8:**
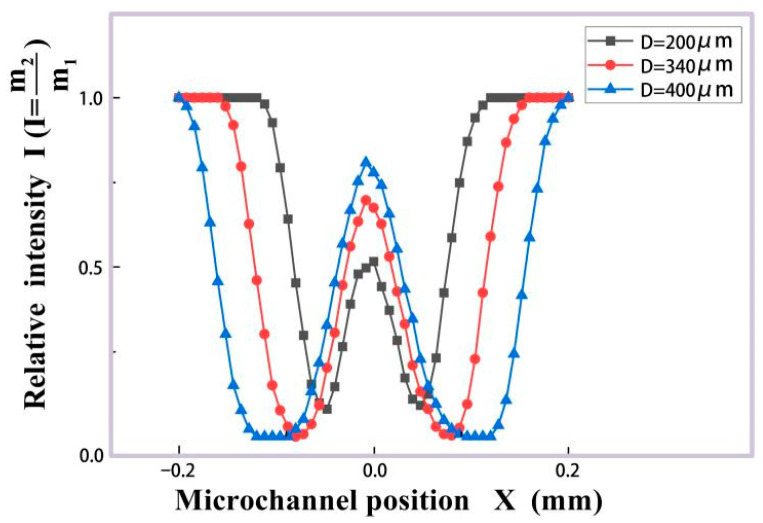
Relative intensity of different microdroplets as they pass through the laser beam.

For microdroplets of different sizes, *D* = 200 μm, *D* = 340 μm and *D* = 400 μm, the relative intensity I from their entering light beam to their leaving was simulated, as shown in Figure 8.

## 3. Experimental Results and Discussion

**Figure 9:**
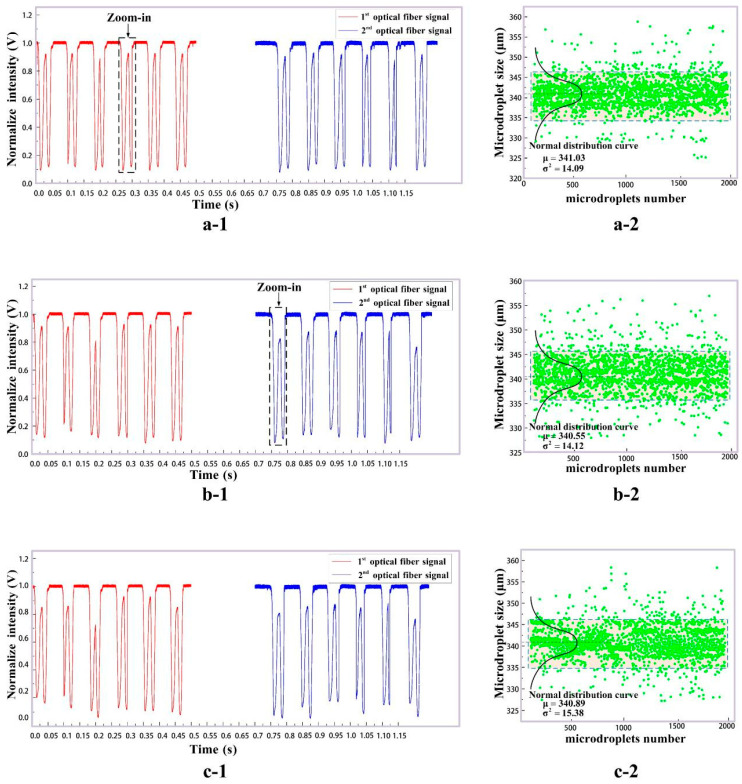
Microdroplet detection pulse signals and their size distribution. (**a-1**) Part of the pulse signals of the first group; (**a-2**) The size distribution of the first group of micro-droplets, each green dot represents one sample; (**b-1**) Part of the pulse signals of the second group; (**b-2**) The size distribution of the second group of micro-droplets, each green dot represents one sample; (**c-1**) Part of the pulse signals of the third group; (**c-2**) The size distribution of the third group of micro-droplets, each green dot represents one sample.

**Figure 10:**
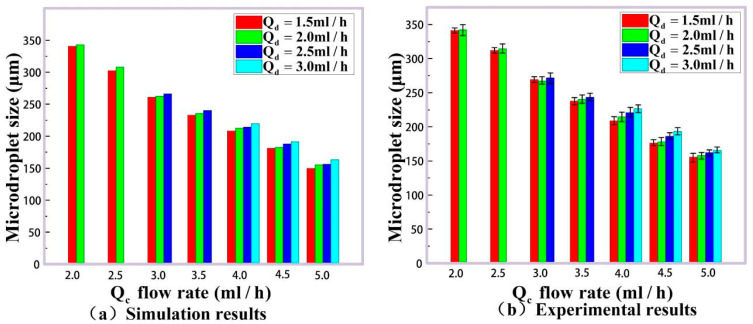
Microdroplets of different sizes are generated by changing the rate of dispersed phase flow. (**a**) COMSOL simulation results of microdroplet size. (**b**) The statistics of microdroplet sizes measured by our experimental method.

Three groups of experimental results, detected by our system in Section 2, are given in Figure 9 with the dispersed phase flow rate $Q_{d}$ = 1.5 mL/h and the continuous phase flow rate $Q_{c}$ = 2 mL/h. According to the COMSOL simulation, the theoretical size of generated microdroplets is D = 340 μm. In each group, 2000 generated microdroplets were detected. Figure 9(a-1,b-1,c-1) are the original pulse signals. The red and blue pulse signals in Figure 9(a-1,b-1,c-1) are collected by optical fibers F3 and F4 in Figure 1, respectively. The red and blue pulses for only six microdroplets in each group are shown as examples. The amplified pulse for a single microdroplet can be found in the zoom-in area of Figure 9(a-1,b-1). Double photoresist method pulses (red and blue pulses) as time-sequence signals can be applied to track the size, velocity and frequency of the generated microdroplets. The abscissa of Figure 8 is the microdroplet’s position inside the microchannel, and that of Figure 9 is the detection time. According to the simulation results in Figure 6b, which show that microdroplets pass through the detection area with the same flow velocity, the abscissas of Figure 8 and Figure 10 thus exhibit a linear relationship. Therefore, both red and blue experimental photoresist method pulses in Figure 9 agree with the TracePro. simulation result for the microdroplets with size D = 340 μm in Figure 8 (the red dotted line). Figure 9(a-2,b-2,c-2) are the statistical analysis for size distributions of all the experimental microdroplets (2000 samples for each group). It can be seen that the generated microdroplet size in the experiments obeys the normality distribution, with the mean value D = 340 μm.

**Figure 11:**
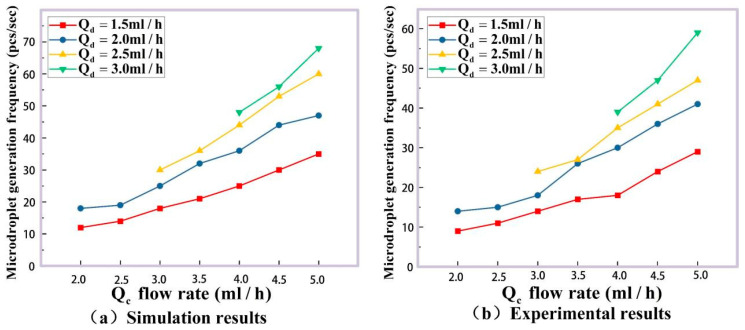
Take the dispersed phase and change the frequency of microdroplet formation when the flow rate of continuous phase is changed. (**a**) The generation frequency of COMSOL simulation results. (**b**) Our experiments results.

Furthermore, a series of experiments with different rates of the dispersed phase $Q_{d}$ and continuous phase $Q_{c}$ are performed to verify the accuracy of our method. The microdroplet generated simulation results for their size and frequency are shown in Figure 10a and Figure 11a, and the experimental detection results by double photoresist method pulses time-sequence tracking method are given in Figure 10b and Figure 11b. The dispersed phase rate $Q_{d}$ is 1.5 mL/h, 2.0 mL/h, 2.5 mL/h and 3.0 mL/h, and the continuous phase rate $Q_{c}$ is 2.0 mL/h, 2.5 mL/h, 3.0 mL/h, 3.5 mL/h, 4.0 mL/h, 4.5 mL/h and 5.0 mL/h.

**Figure 12:**
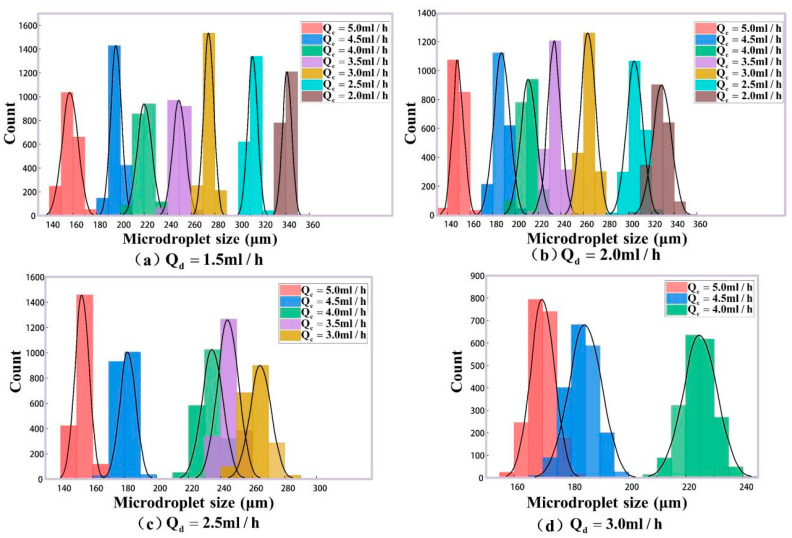
Microdroplet size distribution at different two-phase flow ratios. (**a**) When $Q_{d}$ = 1.5 mL/h, the flow rate of the continuous phase is changed, and the droplet size distribution is obtained. (**b**) When $Q_{d}$ = 2.0 mL/h, the flow rate of the continuous phase is changed, and the droplet size distribution is obtained. (**c**) When $Q_{d}$ = 2.5 mL/h, the flow rate of the continuous phase is changed, and the droplet size distribution is obtained. (**d**) When $Q_{d}$ = 3.0 mL/h, the flow rate of the continuous phase is changed, and the droplet size distribution is obtained.

Figure 10a shows the theory sizes of generated microdroplets according to the phase rates above. And 2000 microdroplets in each group (totally 22 groups) are detected in our experiments for these phase rates. Their mean size values for every group are listed as the column values in Figure 10b, and the size error bars for 2000 microdroplets are labeled on the top of the columns. In order to analyze the experimental size distributions for microdroplet generation in detail, the size histograms for different phase rates are plotted in Figure 12. It can be seen that the data in every group satisfies the normal distribution well. In the meantime, microdroplet generation frequencies in the experiments agree with their simulation results in Figure 11. For further quantitative analysis, our experimental method for the size detection of these generated microdroplets are compared with the classical imaging method [37], as shown in Table 2.

**Figure 13:**
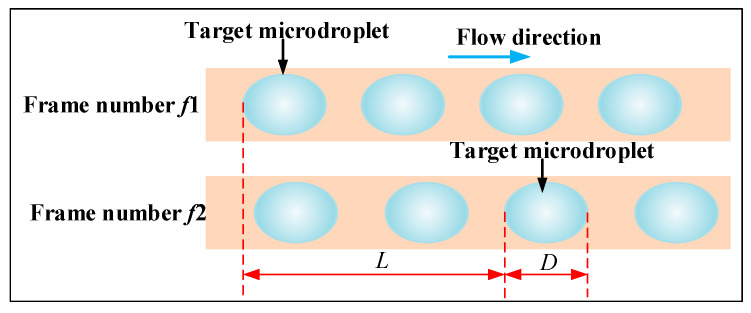
The calculation principle of the microdroplet velocity imaging method.

Among various microdroplet detection methods, the classical imagining method is generally regarded as one of the more accurate techniques due to the use of high-precision cameras and other equipment. Therefore, in order to evaluate the detection accuracy of the dual-photoresistive pulse time-sequence microdroplet detection method proposed in this study, the experiment compared the results with those obtained using the classical imagining method. The principle of the imagining method used in the study to measure microdroplets is illustrated in Figure 13. Here, L represents the distance traveled by the target microdroplet within the microchannel over a certain period, and D denotes the size of the microdroplet. Using an sCMOS (scientific CMOS) camera to record the motion of the microdroplet in the microchannel, images of the microdroplet movement from frame *f*_1_ to frame *f*_2_ can be obtained. Thus, the microdroplet size D can be determined by analyzing the number of pixels corresponding to the microdroplet in any single frame. The specific calculation of the size value *D* is given by the following formula:

(5) $$\frac{n_{w}}{n_{d}}=\frac{500\;\mu m}{D}$$

In Equation (5): *n_w_* represents the number of pixels of the microchannel width; *n_d_* represents the number of pixels of the micro-droplet.

According to the following formula, the velocity *v_img_* of the microdroplets in the microchannel can be derived based on the frame numbers *f*_1_, *f*_2_ of the microdroplet images and the sampling camera frame rate *f_r_*:

(6) $$v_{img}=\frac{f_{r}}{f_{2}-f_{1}}L$$

In Equation (6): *f_r_* represents the frame rate of the camera; *f*_1_ and *f*_2_ are as shown in Figure 13.

In the measurement experiment, in order to obtain the movement images of the microdroplets inside the PTFE tube, an sCMOS scientific-grade camera with a resolution of 2048 × 2048, a pixel size of 6.5 μm and a maximum sampling frequency of 74 FPS (Frame Per Second) was used to collect the micro-droplet images. Finally, the movement speed of the microdroplets was calculated.

For each group, 2000 microdroplets were measured by both the imaging method and our method, as shown in Table 2. The mean size is the average detection value. $D_{E}$ and $D_{I}$ are the results by our method and the imaging method. The maximum error $\left|\delta \right|$ (|δ| = $\frac{\left|D_{E}-D_{I}\right|}{D_{I}}\times 100\%$,) is defined as the maximum value among all the 2000 microdroplets. Across a total of 22 groups of comparative experiments, the maximum error among 44,000 microdroplets was only 1.42%.

Figure 9(a-1,b-1,c-1) show three groups of pulse signals collected with a dispersed phase flow rate = 1.5 mL/h and a continuous phase flow rate of $Q_{c}$ = 2 mL/h; its horizontal coordinate is the time, and the vertical coordinate is the normalized light intensity; (a-2) to (c-2) are the statistic results of the corresponding size. The black curves indicate the Gaussian distribution of the microdroplet size.

Figure 10 shows that microdroplets can only be generated when the two-phase flow rate ratio satisfies certain conditions. So, some of the two-phase flow rate ratios in the figure will not generate microdroplets, and the corresponding microdroplet sizes cannot be shown (e.g., the case when $Q_{d}$ = 1.5 mL/h and $Q_{c}$ = 2.0 mL/h).

**Figure 14:**
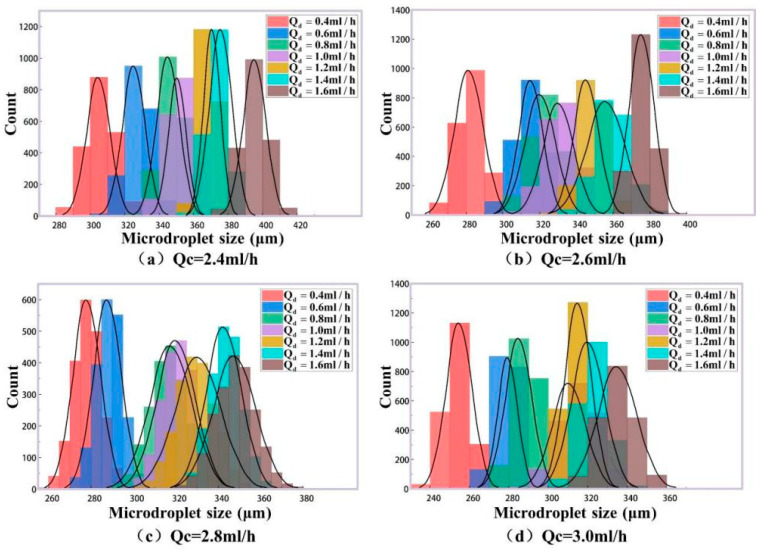
Microdroplet size distribution for different two-phase flow ratios. (**a**) When $Q_{c}$ = 2.4 mL/h, the droplet size distribution is generated by changing the dispersed phase velocity. (**b**) When $Q_{c}$ = 2.6 mL/h, the droplet size distribution is generated by changing the dispersed phase velocity. (**c**) When $Q_{c}$ = 2.8 mL/h, the droplet size distribution is generated by changing the dispersed phase velocity. (**d**) When $Q_{c}$= 3.0 mL/h, the droplet size distribution is generated by changing the dispersed phase velocity.

**Figure 15:**
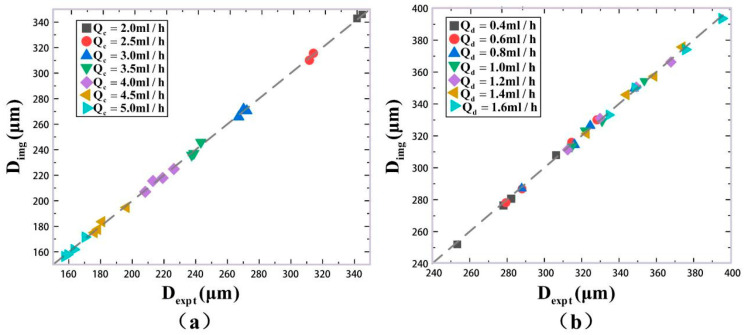
Comparison of microdroplet sizes measured by our experimental method with the image processing method. (**a**) When the dispersed phase $Q_{d}$ = 1.5 mL/h, 2.0 mL/h, 2.5 mL/h and 3.0 mL/h, the continuous phase flow rate was changed. (**b**) The experimental results were obtained by changing the flow velocity of the dispersed phase when the continuous phase was $Q_{c}$ = 2.4 mL/h, 2.6 mL/h, 2.8 mL/h and 3.0 mL/h.

In the above experimental results, the different phase rates were set with the changing of the continuous phase rate $Q_{c}$ and the constant dispersed phase rate $Q_{d}$. This case is called Case A. For example, in Figure 12a, the size distribution of generated microdroplets was analyzed as $Q_{c}$ changed from 2.0 mL/h to 5.0 mL/h, with $Q_{d}$ remaining constant and equal to 1.5 mL/h. Using the same analytical methods, the size distributions with the changing of $Q_{d}$ and the constant value $Q_{c}$ were discussed. It is called Case B. In Figure 14, when $Q_{c}$ is a constant, the size distributions of generated microdroplets with different $Q_{d}$ (0.4 mL/h, 0.6 mL/h, 0.8 mL/h, 1.0 mL/h, 1.2 mL/h, 1.4 mL/h and 1.6 mL/h) were studied. A total of 28 groups of microdroplets with different sizes were generated. For each group, there were 2000 microdroplets. In total, 56,000 microdroplets were detected by our method. Their quantitative analysis compared with classical imaging method is listed in Table 3. To compare Case A and Case B, Figure 15 was plotted to analyze their measurement accuracy with the classical imaging method. Figure 15a is for Case A, and Figure 15b is for Case B. The horizontal coordinate represents measured size by our experimental method denoted by $D_{expt}$, and the vertical coordinate is that by imaging method denoted by $D_{img}$. The measurement results for both Case A and Case B have small deviation with imaging method. Combining the quantitative analysis in Table 3, it was interesting to find that the maximum error in Case B was only 0.90%, representing an improvement in accuracy by 1.58 times compared to Case A. We guess that there may be two reasons. First, although the same size of microdroplets could be generated by changing any one of the rates of dispersed phase and continuous phase, the velocities of generated microdroplets in microfluid are different, which could affect the accuracy for our method in real-time. Second, the double photoresist method pulses depend on scattering light. In our experiments, the refractive index of the continuous phase is 1.40 and that of the dispersed phase is only 1.33. In addition, the volume of the continuous phase in the microfluid is far more than that of the dispersed phase. The changing of the continuous phase rate in Case A may cause more disturbances for scattering light than that of the dispersed phase in Case B.

Furthermore, the current measurement method is only applicable to systems where water serves as the dispersed phase and dimethyl silicone oil serves as the continuous phase. Further research is needed for other materials. Additionally, the research subjects of this paper are sub-micrometer-sized microdroplets with a size range of 100–500 μm. Microdroplets beyond this size range are not within the scope of discussion of this study.

## 4. Conclusions

In this study, an FWMS by double photoresist method pulses is proposed, which provides a microdroplet generation and synchronous detection method with the advantages of accurate, simple and integration. Microdroplets with size 100–500 µm are generated and measured in real-time by double photoresist method pulses time-sequence tracking. The size, velocity and frequency are detected for totally 100,000 generated microdroplets of Case A (44,000 samples) and Case B (56,000 samples). Compared with the classical imaging method, the maximum size error for Case A and Case B are 1.42% and 0.93%, respectively. Case B is found with great interest that has higher accuracy than Case A. There may be some error sources such as injection discontinuity of stepping injection pump and light interference in the experiments. These problems can be alleviated by improving the experimental environment and using high-quality optical components. In conclusion, FWMS for microdroplets generation and detection provides a simple and integrated approach with high efficiency and precision, which may be prospected in the wide range of applications for microbial culture, chemical research, industrial processes and so on. In addition, compared to other methods, the proposed approach not only demonstrates strong real-time performance and online analysis capability and significantly improving the detection efficiency of microdroplet size and velocity, but also exhibits high detection stability, overcoming drawbacks of existing methods such as the weak real-time performance and low efficiency of image processing, the susceptibility to environmental interference in electrical detection and the complex labeling required in laser-induced fluorescence techniques.

## Figures and Tables

**Table 1:** Parameters of dimethyl silicone oil.

	SI	Parameter
Density	g/mL	0.764
Refractive Index	a.u.	1.40
Vapor Pressure (20 °C)	Mm Hg	5
Viscosity	mm^2^/s	100 ± 8
Freezing Point	°C	−55

**Table 2:** Comparison results of our method and the imaging method.

$Q_{c}$ (mL/h)	$Q_{d}$ (mL/h)	Mean Size by Imaging Method (μm)	Mean Size by Our Results (μm)	Maximum Error $\left|\delta \right|$ (%) for 2000 Samples
2.0	1.5	343.51	341.03	0.68
	2.0	346.77	344.22	0.74
2.5	1.5	309.50	311.94	0.78
	2.0	315.99	313.73	0.73
3.0	1.5	271.93	269.75	0.87
	2.0	265.18	267.29	0.81
	2.5	270.03	272.38	0.74
3.5	1.5	235.47	237.71	0.93
	2.0	236.92	239.09	0.95
	2.5	245.58	243.46	0.88
4.0	1.5	206.57	208.39	0.82
	2.0	215.06	213.29	0.79
	2.5	217.43	219.39	0.88
	3.0	224.42	226.42	0.71
4.5	1.5	174.45	176.39	1.22
	2.0	176.97	178.57	0.92
	2.5	183.05	181.24	0.86
	3.0	194.23	196.59	0.93
5.0	1.5	155.73	157.46	1.03
	2.0	157.15	159.38	0.82
	2.5	161.25	163.37	1.42
	3.0	172.02	169.64	0.97

Note: |δ| = $\frac{\left|D_{E}-D_{I}\right|}{D_{I}}\times 100\%$, $\left|\delta \right|$ is defined as the maximum value among all the 2000 microdroplets.

**Table 3:** Comparison results of our method and imaging method.

$Q_{d}$(mL/h)	$Q_{c}$(mL/h)	Mean Size by Imaging Method (μm)	Mean Size by Our Results (μm)	Maximum Error $\left|\delta \right|$(%) for 2000 Samples
0.4	2.4	308.30	305.88	0.90
	2.6	280.16	282.54	0.82
	2.8	276.00	278.37	0.77
	3.0	251.48	253.74	0.93
0.6	2.4	330.37	327.88	0.84
	2.6	316.56	314.11	0.83
	2.8	286.14	288.48	0.78
	3.0	277.55	279.87	0.79
0.8	2.4	350.00	347.33	0.82
	2.6	326.62	324.09	0.76
	2.8	313.91	316.41	0.83
	3.0	286.17	288.51	0.71
1.0	2.4	355.41	352.77	0.78
	2.6	328.90	331.41	0.75
	2.8	323.82	321.38	0.82
	3.0	312.85	315.28	0.73
1.2	2.4	365.58	368.34	0.79
	2.6	351.38	348.70	0.80
	2.8	331.71	329.21	0.71
	3.0	310.57	313.01	0.85
1.4	2.4	376.00	373.68	0.76
	2.6	356.79	359.27	0.82
	2.8	346.14	343.67	0.81
	3.0	320.70	323.15	0.84
1.6	2.4	393.08	395.33	0.76
	2.6	373.60	375.86	0.78
	2.8	350.47	348.10	0.78
	3.0	332.41	334.92	0.85

## Data Availability

The data that support the findings of this study are available from the corresponding author upon reasonable request.
